# Scoping Review of Machine Learning Techniques in Marker-Based Clinical Gait Analysis

**DOI:** 10.3390/bioengineering12060591

**Published:** 2025-05-30

**Authors:** Kevin N. Dibbern, Maddalena G. Krzak, Alejandro Olivas, Mark V. Albert, Joseph J. Krzak, Karen M. Kruger

**Affiliations:** 1 Department of Pediatrics, University of Nebraska Medical Center, Omaha, NE 68198, USA; 2 Department of Biomedical Engineering, Marquette University, Milwaukee, WI 53223, USAkaren.kruger@marquette.edu (K.M.K.); 3 Motion Analysis Center, Shriners Children’s, Chicago, IL 60707, USA; 4 Department of Computer Science and Engineering, University of North Texas, Denton, TX 76205, USA; 5 Doctor of Physical Therapy Program, Midwestern University, Downers Grove, IL 60515, USA

**Keywords:** machine learning, clinical gait analysis, scoping review, artificial intelligence, quantitative gait analysis, optical motion capture, marker-based gait analysis, explainable AI, XAI

## Abstract

The recent proliferation of novel machine learning techniques in quantitative marker-based 3D gait analysis (3DGA) has shown promise for improving interpretations of clinical gait analysis. The objective of this study was to characterize the state of the literature on using machine learning in the analysis of marker-based 3D gait analysis to provide clinical insights that may be used to improve clinical analysis and care. Methods: A scoping review of the literature was conducted using the PubMed and Web of Science databases. Search terms from eight relevant articles were identified by the authors and added to by experts in clinical gait analysis and machine learning. Inclusion was decided by the adjudication of three reviewers. Results: The review identified 4324 articles matching the search terms. Adjudication identified 105 relevant papers. The most commonly applied techniques were the following: support vector machines, neural networks (NNs), and logistic regression. The most common clinical conditions evaluated were cerebral palsy, Parkinson’s disease, and post-stroke. Conclusions: ML has been used broadly in the literature and recent advances in deep learning have been more successful in larger datasets while traditional techniques are robust in small datasets and can outperform NNs in accuracy and explainability. XAI techniques can improve model interpretability but have not been broadly used.

## 1. Introduction

Marker-based 3D gait analysis (3DGA) is a common and powerful tool for understanding the impact of musculoskeletal and neurological impairments on ambulation [1]. These analyses create feature-rich datasets that describe a triplanar motion for joints from the feet through the trunk [2]. The kinematic data produced by 3DGA are commonly reported as rotations in coronal, sagittal, and transverse planes. These data can be time normalized to 100% of the gait cycle and used to create curves that describe all events during gait from initial contact to toe off, through the swing phase, and back to pre-positioning ahead of the subsequent initial contact. Kinetic and electromyographic data are also commonly collected and provide information about muscle activation patterns, joint moments, and power. Clinical interpretation of these complex datasets is challenging and requires the additional incorporation of clinical examination findings to identify impairments and inform treatment decisions [1]. Clinical examination data may include joint range of motion, strength, presence of contractures, joint alignment, and potentially x-ray measurements [1]. This large volume of data presents both a significant burden for clinicians to integrate and an opportunity for improved identification of features relevant to care.

Machine learning (ML) can be applied to improve understanding of these feature-rich 3DGA datasets and augment clinical interpretation [2]. ML techniques have been used to extract or identify relevant features through dimensionality reduction and classification while unsupervised learning has facilitated the discovery of critical clinical gait patterns and important features for diagnosis through clustering [3,4,5]. ML has come into increasing use in both the collection and evaluation of gait analysis data where the literature has established the utility of ML to aid understanding of gait in cerebral palsy [3,6], myelomeningocele [7], post-stroke [8], Parkinson’s disease [9], autism [10], and other musculoskeletal and neurological problems [8,11]. Such studies have focused on detecting gait events, pathological gait, classifying subtypes of disorders, and directly aiding clinical decision making [6,12].

Deep learning (DL) as a subset of ML holds further promise in the analysis of marker-based 3DGA given its ability to provide additional non-linear insights into gait deviations, identification of pathology, and prediction of outcomes [1,13,14]. However, DL typically requires large sample sizes for high-dimensional datasets like these and can have problems with interpretability. Additionally, DL does not necessarily outperform classical ML methods. Thus, the purpose of this study was to understand both how machine learning and deep learning have been successfully deployed in the analysis of marker-based gait data to aid in the interpretation of these data. Specifically, we conducted a broad scoping review of the 3DGA literature to capture which techniques have been utilized to improve both supervised and unsupervised differentiation of these complicated time series data. The specific goals of this project were to identify which ML techniques have been utilized in the evaluation of 3DGA data, whether and how they evaluated whole time series data, and to identify gaps where rapid advancements in deep learning can be used to improve understanding of 3DGA datasets.

### Related Literature Reviews

Given the proliferation of machine learning techniques in quantified gait analysis, recent reviews have focused on the use of deep learning for quantified gait analysis [13,15,16]. These studies have provided reviews of between 43 and 63 articles where the focus has equivocated analysis of data from marker-based 3DGA, inertial measurement units (IMUs), accelerometers, plantar pressures, force plates, electromyography, and gait video sources. The number of studies focused on marker-based data are not directly reported in two of these [15,16]. However, a comprehensive background on deep learning algorithms has been detailed in a 2024 systematic review on gait analysis in cerebral palsy and stroke along with a report of 31 studies using optoelectronic (marker-based) 3DGA [13]. Their goal was understanding the main challenges in transferring proposed machine learning methods to clinical applications [13]. They identified compromised reliability and practicality of most studies due to insufficient justification for feature selection and ML algorithms as well as datasets that do not contain representative populations.

The primary contributions of this study, in contrast to these related reviews, are to the following:

(1) Provide a focused review of all machine learning techniques, not exclusively deep learning, used in the analysis of gold standard marker-based 3DGA for supervised and unsupervised learning.
(2) Trends in the use of different ML techniques and comprehensive reporting of the strengths and deficiencies of each method.
(3) Discussion of clinical relevance and opportunities for future research.

The analyses presented in this scoping review offer a broader ability to see where current deep learning techniques fit in the literature. While trends toward deep learning continue, traditional machine learning techniques may be overlooked despite benefits in small datasets, ease of implementation, and interpretability.

## 2. Materials and Methods

The Preferred Reporting Items for Systematic Reviews and Meta-Analyses extension for Scoping Reviews (PRISMA-ScRs) was utilized to guide the creation of this study [17]. The search was completed without protocol registration between the dates of 1 January 2000 and 21 July 2023. The search results were loaded into Rayyan.ai to facilitate the detection and removal of duplicate results as well as collaboration between reviewers.

### 2.1. Eligibility Criteria

The search included the PubMed and Web of Science databases on the topic of using machine learning to classify or cluster the marker-based 3DGA data. An initial collection of eight articles relevant to the topic was identified by the team of co-authors to help guide the creation of search terms [3,5,6,8,9,10,11,12]. Terms were modified with keywords from the articles to generate the overall strategy and applied to both the PubMed and Web of Science databases. The search terms and strategy were refined until all 8 articles were captured in the review. The full strategy can be found in Appendix A.

### 2.2. Selection of Evidence

The titles and abstracts of all articles were screened by three reviewers into the categories of include, reject, and maybe. Articles were considered based on the criteria developed by the co-authors to gather works relevant to the purpose of this study in using machine learning on 3DGA to aid clinical classification and decision making.

There were three critical components that were used to define the eligibility of articles in the screening process, as follows:

1. Use of marker-based 3D gait analysis data.
2. Analysis of data by machine learning techniques.
3. Leveraging of machine learning techniques to make predictions or classifications.

The third screening criterion was potentially the most subjective and thus left more open to individual interpretation during the abstract screening process with an option to select “maybe” for inclusion in the initial screening. The final criteria were based on whether analysis used machine learning to specifically aid clinical decisions or delineate groups via unsupervised (clustering) and supervised (classification) methods. The exclusion criteria included the non-peer reviewed literature, studies without machine learning methodologies, studies that did not use opto-electric/marker-based technology for 3DGA, studies with insufficient data, and studies not available in English.

### 2.3. Synthesis of Results

Data items included the number of participants, date of publication, clinical population studied, and specific machine learning methods used to make predictions, clusters, or classifications on 3DGA data. As many machine learning articles employ and report varied methods, only the best method, specifically the method with the greatest accuracy as reported by the authors of each article, was documented. The years in which methods have been used were also quantified to identify both recent trends and enduring methods. The clinical populations studied are reported to understand where ML in gait analysis has been used. Finally, a summary of which ML methods have been used in combination with the number of participants and the year published was created to enhance understanding of where progress has been made and on what types of datasets. The number of participants rather than the number of data collections was used to provide a clearer understanding of the scope of the studies and generalizability of their methods.

## 3. Results

**Figure 1:**
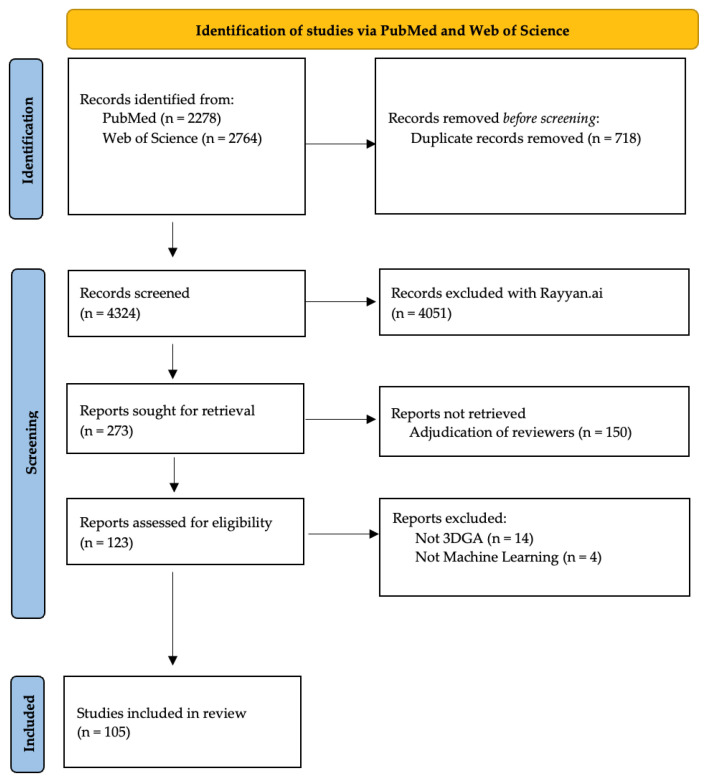
Selection process undergone for studies in the review adapted from the PRISMA flow diagram [17].

A total of 4324 articles were reviewed for potential inclusion after removing duplicates (n = 718) from the PubMed (n = 2278) and Web of Science (n = 2764) searches (Figure 1). The adjudication of the 273 abstracts identified for potential inclusion by the reviewers resulted in 123 full-text articles reviewed for eligibility. The final review excluded 18 articles, 14 for non-marker-based techniques and 4 for not using machine learning to evaluate the data. This left us 105 full-text articles to report the machine learning techniques utilized and clinical conditions observed. The techniques are described in detail below and the number of participants and average year of publication are detailed for each method.

### 3.1. Distribution of Clinical Conditions

**Figure 2:**
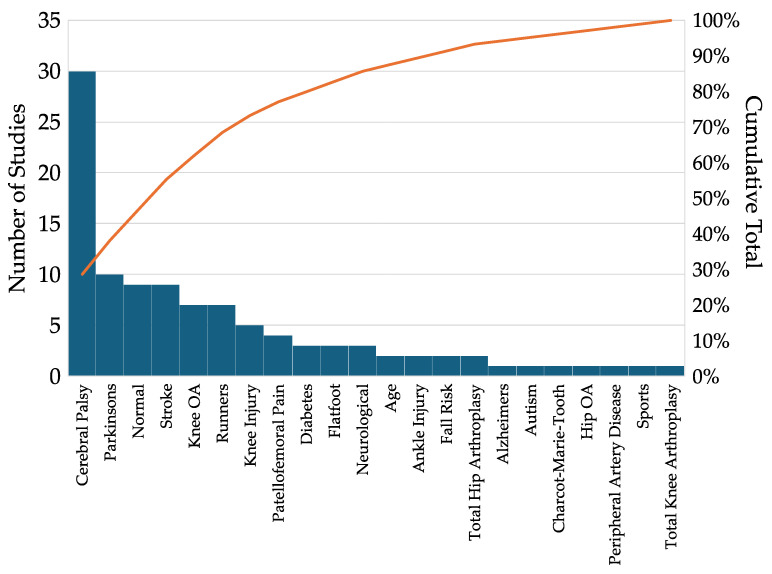
Distribution of clinical conditions studied in 105 articles evaluated.

There were 22 clinical conditions evaluated by the studies in this review (Figure 2). Cerebral palsy (n = 30) was by far the most reported condition evaluated followed by Parkinson’s disease (n = 10) and post-stroke (n = 9) evaluations. Other conditions with at least three studies included normal conditions explicitly reported as without pathology (Normal, n = 9), Knee Osteoarthritis (Knee OA, n = 7), runners (n = 7), ligamentous knee injury (Knee Injury, n = 5), patellofemoral pain (n = 4), diabetes (n = 3), flatfoot (n = 3), and combined evaluation of neurological conditions (Neurological, n = 3).

### 3.2. Machine Learning Techniques

**Figure 3:**
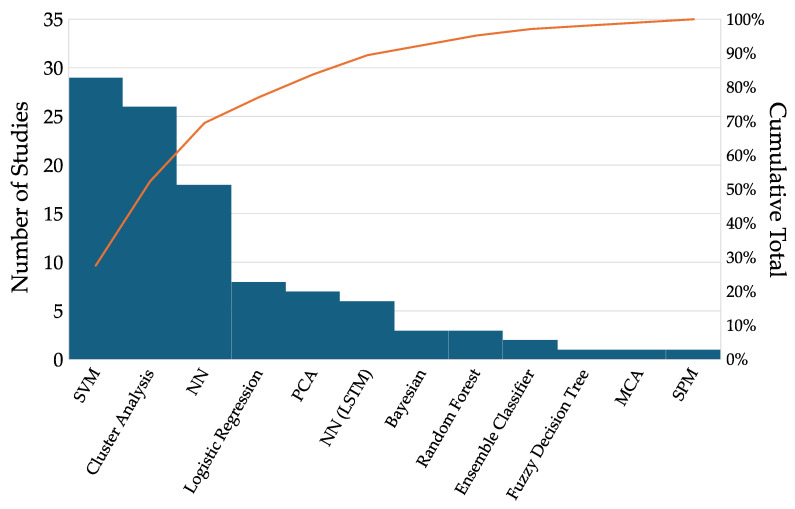
Distribution of machine learning and statistical techniques used. (Acronyms: support vector machine (SVM), neural network (NN), Principal Component Analysis (PCA), Long Short-Term Memory, statistical parametric mapping, multiple correspondence analysis (MCA)).

There were several primary ML techniques utilized (Figure 3). The largest number of manuscripts found support vector machines (SVMs, n = 29) to be the most effective at tackling their classification challenges with clinical gait data. Cluster analyses after PCA data reduction were the second most common (Cluster Analysis, n = 26), followed closely by neural networks (NNs, n = 18) and logistic regression (n = 8). We separate Long Short-Term Memory NNs (LSTM, n = 6) as they directly analyze the full time series data rather than the extracted features and warrant separate discussion. Other techniques were classical Bayesian (n = 3), random forest (n = 3), ensemble (n = 2), fuzzy decision tree (n = 1), multiple correspondence analysis (n = 1), and statistical parametric mapping (n = 1).

### 3.3. Support Vector Machines (SVMs, n = 29)

**Figure 4:**
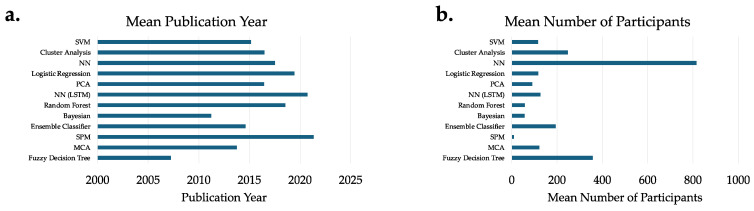
(**a**). Mean publication year for each study separated by primary machine learning analysis technique. (**b**). Mean number of participants used in each study. (Acronyms: support vector machine (SVM), neural network (NN), Principal Component Analysis (PCA), Long Short-Term Memory, statistical parametric mapping, multiple correspondence analysis (MCA)).

SVMs are valued for their ability to define flexible boundaries, or hyperplanes that optimally separate data. On small datasets with high dimensional data, as seen in this review (average sample size n = 116, Figure 4b), SVMs were successful, even outperforming neural networks [11]. Interestingly, the SVM was also used in combination with an NN to improve classification accuracy, suggesting that ensemble approaches may benefit from combining the structure of SVM and flexibility of NN [8]. SVMs were used in the analysis of aging [5,18,19], arthritic gait [20,21,22,23,24,25], autism[10], cerebral palsy [26,27], runners [28,29,30,31,32], Parkinson’s disease [33,34], knee injury [11,35], patellofemoral pain [36,37], neurological [38], post-stroke [8], and normal conditions (control or otherwise explicitly without pathology) [39,40,41,42].

### 3.4. Cluster Analysis (n = 26)

Cluster analysis encompasses a set of traditionally unsupervised machine learning techniques that identify natural separation within the data. These studies had the third largest average sample size (n = 247, Figure 4b). Principal Component Analysis (PCA) was commonly used for dimensionality reduction to perform clustering. Nearly half the cluster analyses were performed in populations with cerebral palsy [3,43,44,45,46,47,48,49,50,51,52,53], differentiating different levels and types of impairment. It was also used to evaluate Charcot–Marie–Tooth [54], diabetes [55,56], flatfoot [57,58], knee arthroplasty [59], knee arthritis [60], patellofemoral pain [61], post-stroke [62,63,64], Parkinson’s disease [65,66], and normal [67] conditions.

### 3.5. Neural Networks (NNs, n = 18)

Neural networks serve as a foundational tool for deep learning and have been used with increasing frequency. These were utilized in the largest datasets (average sample size n = 815, Figure 4b). NNs are well described throughout the literature so this review of the 24 articles will focus on how and in what unique ways it has been applied in the analysis of marker-based 3DGA. They have been used most frequently in the analysis of cerebral palsy [12,68,69,70,71,72] and Parkinson’s gait [9,73,74,75,76,77,78], but have also been used in post-stroke [8,79,80], flatfoot [81], normal [82,83], knee injury [84,85], and peripheral artery disease [86]. Studies primarily used convolutional NNs, and LSTM networks are discussed separately.

### 3.6. Long Short-Term Memory NN (NN (LSTM), n = 6)

Long short-term memory (LSTM) is a type of Recurrent Neural Network (RNN) that is particularly well suited to the task of analyzing time series data from 3DGA and has more recently come into use (Figure 4a). It was utilized in more modestly sized datasets than other NNs (average sample size n = 124, Figure 4b) in Parkinson’s disease [73], post-stroke [87], detecting ankle injuries [88,89], and cerebral palsy [12,72]. RNNs, unlike traditional neural networks, effectively enable the processing of data sequentially by retaining the memory of previous inputs. A challenge in traditional RNNs is that they can fail to connect distant “long-term” dependencies, which leads to a “vanishing gradient” where data from earlier in a sequence can be lost. LSTM bolsters these long-term connections by incorporating “memory cells” and “gates” to selectively retain and forget information. This mitigates the vanishing gradient problem, allowing for deeper networks to be trained. Critically for gait analysis, this can enable the use of full time series information in model training. This improved the detection of gait events and classification without significant effort expended in manual feature extraction.

### 3.7. Other Machine Learning Techniques (n = 26)

Traditional techniques like logistic and linear regression [90,91,92,93,94,95,96,97,98], PCA [91,97,99,100,101,102,103,104], multiple correspondence análisis [105], along with Bayesian [106,107,108], fuzzy decision tree [109], random forest [6,110,111,112,113], and ensemble classification [99,100] were also found and are well described in the literature. We highlight ensemble classifiers again here as they can combine machine learning techniques to improve model performance by overcoming the weaknesses of individual models. This can lead to more robust predictions with greater accuracy that are more generalizable to new data. As described above, SVM and NN, two of the most powerful and popular methods, were combined to improve performance in Cui et al.’s work, classifying hemiparetic stroke[8]. Although not a classification or clustering technique, we also captured statistical parametric mapping (SPM) as a recently utilized (Figure 4a) powerful tool for discriminating the significance of differences in time series 3DGA data between groups [114].

### 3.8. Explainable AI (XAI)

While techniques like decision trees and linear methods are inherently explainable, studies using neural networks or ensemble methods make predictions without revealing how the predictions are made. These “blackbox” models have potential performance advantages over traditional ML, but are limited in their clinical applicability as healthcare providers cannot interpret why decisions are made to assess potential errors and biases. Thus, XAI methods have been developed to highlight the features that ML models use to make predictions. This offers great potential to enhance the clinical integration of high-performing ML and DL models.

### 3.9. Shapley Additive Explanations (ShAPs)

ShAP analyses enable interpretability of the relative importance of model input features toward both classification and regression problems. More specifically, a ShAP dataset can be used to reproduce a machine learning model’s non-linear prediction with a linear sum of the ShAP values plus a constant to obtain a prediction. In Kokkotis et al.’s 2022 paper evaluating gait parameters associated with anterior cruciate ligament injury, ShAP values were leveraged to characterize the relative importance of 26 different gait biomechanical parameters in classifying controls, ACL-deficient, and ACL-reconstructed knees [11]. Additionally, explanations between each pair of classes were performed to identify which features were relevant to predictions. This presents data in a similar manner to traditional regression models, where the direct combinations of variables and their weighted importance to the model are quantified.

### 3.10. Local Interpretable Model-Agnostic Explanations (LIMEs)

Similar to ShAP, LIME provides interpretability through approximation of the model with a simpler interpretable model. In contrast to ShAP, which can explain the overall model, LIME focuses on localized explanations by evaluating the changes in model outputs by perturbing inputs. This results in a map of input feature relevance that does not have an overall direct linear model correlate. As this general framework is model agnostic, it enables use with both traditional and deep learning techniques. However, as LIMEs are seeded from random initial conditions and the underlying model may not be stable around all local points, it is necessary to evaluate the stability of explanations. This is particularly relevant to gait analyses with high input feature dimensionality. Thus, studies should adopt strategies for countering variability in LIME results [68]. While LIME was mentioned in our review, it was not utilized for explanations [14].

### 3.11. Layer-Wise Relevance Propagation

Filtjen et al. described LRP as an attributional technique that decomposes the prediction of an output computed over a gait cycle into relevance scores that can be mapped onto each input feature [9]. Functionally, this is achieved by making a prediction based on the input data through the standard forward pass through an NN, and propagating the prediction backward layer by layer using specific rules to enhance an explanation that is equivalent to an otherwise noisy Gradient × Input. Filtjen et al. used the Epsilon Rule that results in sparser explanations that are less noisy. The result is a map of input features that are important to the model for identifying each pathology; in their case, the freezing of gait in Parkinson’s disease [9].

## 4. Discussion

Machine learning has shown promise to integrate and analyze the complex datasets generated by 3DGA, but has yet to make the transition to common clinical utilization. Recent rapid advancements in ML techniques may help to bridge this gap. This scoping review sought to understand the state of the literature on the use of machine learning in marker-based clinical gait analysis toward the identification of promising techniques that can be used to enhance clinical care with ML. It characterized the techniques used in primarily clinical populations on these gold standard marker-based 3DGA datasets, the size of the datasets, and the years they were published. In this manner, the evolution and success of different machine learning techniques in the analysis of quantified gait data were characterized. Despite the large volume of studies captured, we did not find any studies directly evaluating the clinical integration of data from machine learning into workflows. While we found an expected and clear trend toward the utilization of deep learning techniques and NNs in the literature, traditional clustering and classification methods were frequently used, and as noted previously, successful in the analysis of modestly sized datasets. Thus, this review serves as a reminder of the utility of more traditional machine learning techniques like regression, SVM, and simple clustering, particularly in smaller datasets where they can identify robust explainable relationships that can more readily enhance clinical understanding.

Of the studies evaluated, there was substantial variability in the number of participants reported for each selected machine learning technique. This potentially reflects both the reporting methodology used in each paper and the utility of each technique with different amounts of data, sample sizes, and features. The literature has provided some recommendations for the sample size in different techniques. For PCA, 5 to 10 samples per feature are recommended [115,116], while the SVM recommendations are 10–20 samples per feature [117], and neural networks use 100 samples per feature [118]. To test whether the sample size is adequate for each particular problem, a variety of strategies may be employed. For PCA, Bartlett’s test of sphericity or the Kaiser–Meyer–Olkin tests may be used. For SVM and other machine learning methods, power analyses, learning curves, and robust cross-validation are warranted to create robust generalizable models. If unreported, it may be assumed that the machine learning techniques reported would have performed best on the dataset. However, articles may only report one method but test several. Therefore, our results are likely limited by recency bias toward neural networks and deep learning techniques due to their emergent popularity. It is worth noting that reporting of these preliminary findings, particularly in smaller datasets, may better place new developments in the context of true advancements for clustering and classification problems, as well as provide more explainable, clinically relevant insights.

Despite the increased use of machine learning in 3DGA, very few studies in our review focused on or evaluated the implementation or integration of ML models in a clinical setting. Only two articles proposed the use of machine learning in a direct decision support capacity [6,33]. Importantly, it is in this capacity that AI and ML are most readily able to be employed by healthcare providers. Since 1st August in the European Union, AI must now comply with the European Artificial Intelligence Act [119]. This ensures that the AI-based software used for medical purposes has appropriate risk mitigation, high-quality datasets, clear user information, and human oversight. The FDA provides similar non-binding recommendations for the use of ML to support making predictions, recommendations, or decisions [120]. Thus, ML for medical use requires the integration of data by a healthcare professional with a support framework like the one described by Chia et al. or Schwartz et al. [2,6]. Specifically, an optimal support framework may provide clinicians with potential diagnoses, relevant impairments, treatment recommendations, and outcome prediction. Critically, it should also identify why and with what confidence these ML systems make predictions. Chia et al. explained that model confidence can improve clinical decision making through reinforcement or reconsideration of decisions based on agreement with high confidence model results [6]. Additionally, they proposed using explainable ML methods to empower clinicians to examine the reasoning underlaying predictions. Therefore, before ML models can be clinically useful, they must demonstrate both their utility identifying problems in concert with healthcare professionals and the interpretability of their logic to ensure the results are without error or bias.

Adapting the ML models reported in this study to conform to these standards may contribute to solving problems presented in recent clinical practice guidelines, such as the following: using 3DGA to predict surgical outcomes, identifying combinations of discrete variables that inform surgical decisions and improve outcomes, characterizing the importance of 3DGA features and the role of supplemental data, and the recognition of gait deviations from the entire gait cycle rather than discrete kinematic variables [1]. Several studies identified in this review already align well with these priorities and can serve as a foundation for more translational research once integrated into a framework with human oversight. Specifically, surgical outcomes were predicted using PCA and the multiple linear regression of a pre-operative physical exam and the 3DGA data combined with surgical codes by Galarraga et al. [97]; how specific gait features informed surgical decisions were explored by Chia et al. [6]; and the use of full gait cycle data to identify gait deviations was further explored by papers using LSTM networks [12,72,73,87,88,89] and by Filtjens et al. [9] using CNNs. While these studies provide an excellent foundation toward achieving clinical translation, more research with larger high-quality 3DGA datasets is needed to evaluate clinical benefits and achieve clinical practice integration. Recent work with markerless 3DGA and other technologies may help increase the size of datasets toward these goals, but care should be taken to validate these methods and to identify if additional variability introduced by alternative methods hinders potential clinical insights [121,122].

Toward improving the understanding of ‘blackbox’ machine learning models of gait data, several recent studies have proposed the use of explainable-AI (XAI) techniques to aid in the clinical interpretation of classification and clustering predictions created on these complicated time series data. These methods can directly highlight input features that contribute to outcome prediction on whole datasets or individual predictions. This is useful for bridging the gap between ML and clinical insight, as it can identify which specific time points and deviations contribute to ML predictions of outcomes or classifications. Shapley Additive Explanations (ShAPs), Local Interpretable Model Explanations (LIMEs), and Layer-Wise Relevance Propagation (LRP) were all identified and reviewed within this work. ShAP and LIME provide an estimation of input feature importance by perturbing input features and observing effects on the model output. ShAP leaves each feature out to observe its impact on the model, while LIME slightly modifies each feature to observe its impact. They may be useful as they can be implemented on any machine learning model because they only manipulate inputs and observe outputs, agnostic to the model used, and highlight the relevance of input features for specific predictions. Additionally, ShAP can be used to explain whole model behavior as well as individual predictions. LRP is also flexible in its prediction of individual and global predictions, but works by propagating outputs back to inputs through neural networks, enabling more efficient computation. Slijepcevic et al. used another propagation-based technique, Grad-CAM, in the evaluation of cerebral palsy gait classification [123]. Grad-CAM uses the gradients of the final convolutional layer, resulting in coarser but potentially more interpretable explanations compared to LRP, which assigns relevance to individual neurons that provide more detailed but also more complex explanations. In the context of 3DGA, this roughly means that Grad-CAM looks at the impact of portions of the gait cycle like mid-stance rather than individual time points. Interestingly, the same paper found that random forests out-performed deep neural networks and attributed the result to the superior performance of “traditional” machine learning techniques and their ability to generalize well with small training samples. Another recent paper by Özateş et al. used LIME to explain the results of an ensemble classifier for foot conditions [124]. They further highlighted the utility of traditional machine learning techniques and the importance of feature extraction in limited datasets. This aligns with a finding of our review that the inclusion of expert knowledge in feature extraction substantially improves models [90]. When deep learning was attempted, some papers additionally reported success in augmenting datasets to enable more appropriate training with less overfitting [88,89].

While this review covers techniques that have been reported in the literature, there are other ML methods that may warrant future exploration. Despite recent advances, transformer models like those used in ChatGPT (chatgpt.com, generative pre-trained transformer) have not found their way to analysis of marker-based gait data. These models may hold promise for analysis of larger 3DGA datasets, as their full attention models have performed well with other sequential datasets [125]. However, as discussed above, there are substantial hurdles to overcome with the size of available clinical datasets, specifically their limited number of samples relative to the high dimensionality of their features.

Relatedly, another important limitation to this work is that it chose to evaluate only marker-based 3DGA. This choice likely precludes analysis of more cutting-edge deep learning techniques on larger time-series datasets that may also improve analysis in marker-based 3DGA. Yet, this scoping review explicitly focused on marker-based 3DGA for two primary reasons. The first and most important reason is that quantitative clinical gait analyses frequently rely on this gold standard technology [121]. Thus, the efficacy of different machine learning methods in aiding classification or clustering of these data was captured in this review, and was more likely to be closest to clinical translation. This is evidenced by 89 of the 108 studies (84.7%) being conducted on clinical populations with a goal of improving either the reliability of classification or better characterizing disease and disorder through novel clustering. The second is that there has been a rapid proliferation of video and inertial measurement unit (IMU) gait analysis techniques that rely on these gold standard collections. Indeed, while we did not record the reasons for rejection by each reviewer during the initial screening, there were many examples of papers reporting machine learning’s use to improve video-based gait analyses or inertial measurement unit (IMU) gait data. In these studies, a large focus was placed on the development and validation of these newer and less reliable data against gold standard marker-based 3DGA rather than the advancement of clinical interpretation.

## 5. Conclusions

This scoping review summarizes the rapid progress made toward the use of machine learning in marker-based 3DGA. While machine learning has been used most frequently in cerebral palsy, over 80% of the studies reported analysis of highly varied clinical conditions. The integration of ML models into clinical decision making has not been thoroughly evaluated. Decision support frameworks offer the most direct and feasible path toward clinical integration. In these frameworks, ML models must demonstrate both utility in identifying problems and the interpretability of their logic to clinicians to ensure the results are without error or bias. Recent trends toward the analysis of full time series data with LSTM networks and explainable AI represent significant progress and can facilitate the interpretation of these complex time series data. The use of traditional machine learning techniques is still warranted in the analysis of marker-based 3DGA, demonstrating good performance in smaller datasets and having improved explainability.

## Data Availability

No new data were created or analyzed in this study.

## Abbreviations

The following abbreviations are used in this manuscript:

3DGA	3D Gait Analysis
AI	Artificial Intelligence
ML	Machine Learning
DL	Deep Learning
SVM	Support Vector Machine
NN	Neural Network
RNN	Recurrent Neural Network
LSTM	Long Short-Term Memory
XAI	Explainable Artificial Intelligence
ShAP	Shapley Additive Explanation
LIME	Local Interpretable Model Explanation
LRP	Layer-Wise Relevance Propagation

## Appendix A

This appendix contains the Pubmed and Web of Science search terms used to identify the relevant articles. The search was conducted for articles between the dates of 1 January 2000 and 21 July 2023.

### Appendix A.1. PubMed Search Terms

(“Gait Analysis”[Mesh] OR “Gait”[Mesh] OR “Biomechanical Phenomena”[Mesh] OR “biomechanical” OR “marker-based gait analysis” OR “Kinematics” OR “Kinematic” OR “Kinematic Features” OR “Kinematic Features”[tiab] OR (movement* AND physiolog*) OR “Range of Motion, Articular” OR “articular range of motion” OR “Joint Range of Motion” OR “In vivo kinematic” OR “In vivo kinematics” OR “coordinate system” OR “coordinate systems” OR “dual euler angles” OR “dual euler angle” OR “euler angles” OR “euler angle” OR “helical axis” OR “joint coordinate system” OR “joint coordinate systems” OR “joint kinematics” OR “joint kinematic” OR “motion analysis” OR “motion analyses”) AND (“Machine Learning”[Mesh] OR “Expert Systems” OR “Fuzzy Logic” OR “Deep Learning” OR “Deep-Learning” OR “Supervised” OR “SVM” OR “Support Vector Machines” OR “Neural Networks, Computer” OR “Supervised Machine Learning”[Mesh] OR “Unsupervised Machine Learning”[Mesh] OR “Support Vector Machine”[Mesh] OR “Random forest” OR “Artificial Intelligence”[Mesh] OR “Cluster Analysis”[Mesh] OR “Principal Component Analysis”[Mesh] OR “Explainable AI” OR “Explainable Artificial Intelligence” OR “Explainable Artificial Intelligence”[tiab] OR “Layer-wise relevance propagation” OR “LRP” OR “SHapley Additive exPlanations” OR “shapley additive explanations” OR “SHAP” OR “XAI” OR “Explainable machine learning” OR “Explainable machine learning”[tiab] OR “XML” OR “Counterfactual method” OR “counterfactual” OR counterfact* OR “Generalized additive model” OR “Local interpretable model-agnostic explanations” OR “Local interpretable model agnostic explanations” OR “LIME” OR “graph neural networks” OR “Deep Taylor Decomposition” OR “Prediction Difference Analysis” OR “Testing with Concept Activation Vectors” OR “XGNN” OR “Explainable Graph Neural Networks” OR “Predictive Modeling” OR “Data Mining” OR “Pattern Recognition” OR “Autoencoder” OR “Transformers” OR “Transfer Learning” OR “Intelligent Systems” OR “Automated Reasoning” OR “Causal Reasoning” OR “Generative Models” OR “Feature Importance”) AND (“Gait type” OR “Phenotype” OR Phenotyp* OR “responders” OR “non-responders” OR “decision support system” OR “biomechanical parameters” OR “kinematic foot types” OR “kinematic foot type” OR “kinematic type” OR “Gait Disorders, Neurologic” OR “Gait Disorders” OR “Classification” OR “gait-events” OR “Movement Disorders” OR “Subtype” OR “subtyping” OR “subtypes”) NOT ( robot*)

### Appendix A.2. Web of Science Search Terms

TS = (“Gait Analysis” OR “Gait” OR “Biomechanical Phenomena” OR “biomechanical” OR “marker-based gait analysis” OR “Kinematics” OR “Kinematic” OR “Kinematic Features” OR “Kinematic Features” OR (movement* AND physiolog*) OR “Range of Motion, Articular” OR “articular range of motion” OR “Joint Range of Motion” OR “In vivo kinematic” OR “In vivo kinematics” OR “coordinate system” OR “coordinate systems” OR “dual euler angles” OR “dual euler angle” OR “euler angles” OR “euler angle” OR “helical axis” OR “joint coordinate system” OR “joint coordinate systems” OR “joint kinematics” OR “joint kinematic” OR “motion analysis” OR “motion analyses”) AND TS = (“Predictive Modeling” OR “Pattern Recognition” OR “Autoencoders” OR “Intelligent System” OR “Automated Reasoning” OR “Causal Reasoning” OR “Generative Models” OR “Feature Importance” OR “Machine Learning” OR “Expert System*” OR “Fuzzy Logic” OR “Deep Learning” OR “Deep-Learning” OR “Supervised” OR “SVM” OR “Support Vector Machine*” OR “Neural Networks “ OR “Supervised Machine Learning” OR “Unsupervised Machine Learning” OR “Random forest” OR “Artificial Intelligence” OR “Cluster Analysis” OR “Principal Component Analysis” OR “Explainable AI” OR “Explainable Artificial Intelligence” OR “Explainable Artificial Intelligence” OR “Layer-wise relevance propagation” OR “LRP” OR “shapley additive explanations” OR “SHAP” OR “XAI” OR “Explainable machine learning” OR “XML” OR “Counterfactual method” OR “counterfactual” OR “Generalized additive model” OR “Local interpretable model-agnostic explanations” OR “Local interpretable model agnostic explanations” OR “LIME” OR “graph neural networks” OR “Deep Taylor Decomposition” OR “Prediction Difference Analysis” OR “Testing with Concept Activation Vectors” OR “xgnp” OR “Explainable Graph Neural Networks”) AND TS = (“Gait type*” OR Phenotyp* OR “responder*” OR “non-responder*” OR “decision support system*” OR “biomechanical parameter*” OR “kinematic foot typ*” OR “kinematic foot typ*” OR “kinematic typ*” OR “Gait Disorder*” OR “Classification” OR “gait-event*” OR “Movement Disorder*” OR “Subtyp*”)
